# Systemic Effects of Nitrate, Asparagine, and Glutamine on Nodulation and Biological Nitrogen Fixation in Soybean

**DOI:** 10.3390/plants15081187

**Published:** 2026-04-13

**Authors:** Jixuan Sun, Duo Zhao, Xiaomei Li, Liang Yang, Wei Zhao, Sha Li, Shoukun Dong

**Affiliations:** 1 College of Agriculture, Northeast Agricultural University, Harbin 150030, China; sunjixuan621@163.com (J.S.); 15776291995@163.com (D.Z.); hongyan198125@163.com (L.Y.); zhaoweiscmy@163.com (W.Z.); 2 Heilongjiang Agricultural Engineering Vocational and Technical University, Harbin 150025, China; eileen828@126.com; 3 College of Resources and Environment, Northeast Agricultural University, Harbin 150030, China

**Keywords:** soybean, nitrate, asparagine, glutamine, nodulation and biological nitrogen fixation

## Abstract

Although it is well established that nitrate exerts an inhibitory effect on nodulation and biological nitrogen fixation in soybean, the underlying mechanism remains unclear. In soybean plants, nitrate is assimilated into asparagine (Asn) and glutamine (Gln); their systemic circulation within the plant may contribute to the reduced N-fixation capacity of nodules. To investigate the effects of nitrate, Asn, and Gln on soybean nodulation and biological N fixation, a unilateral nodulated double-root soybean system was used. The non-nodulated side roots were supplied with nitrate (14 mM), Asn (20 mM), or Gln (20 mM), while the nodulated side roots were not supplied with N. Changes in nodule number, nodule dry weight, nitrogenase activity, and N compound content were analyzed after 4 and 10 days of treatment. The results showed that all three exogenous N sources significantly reduced nodule number, nodule dry weight, and nodule nitrogenase activity after both 4 and 10 days of treatment, while promoting the accumulation of ureides, Asn, and Gln in leaves. Nitrate and Asn treatments increased the accumulation of ureides and Asn in nodules, whereas Gln had no significant effect on nitrogenous compounds in nodules. These results suggest that nitrate inhibits nodulation and biological N fixation primarily through its conversion to Asn in soybean plants. The accumulation of Asn in shoots and nodules may suppress nodulation and biological N fixation by promoting ureide accumulation.

## 1. Introduction

Leguminous crops possess a unique ability for biological nitrogen (N) fixation. They fix atmospheric N_2_ to supply their own N nutrition through the formation of nodules in symbiosis with rhizobia. However, biological N fixation by nodules alone cannot fully meet the N requirements for plant growth and development, and the uptake of exogenous N is also essential [1,2]. Nitrate is one of the main forms of N absorbed by legume crops from soil, and its effects on soybean nodulation and biological N fixation are systemic [3]. When nitrate concentration is low, plants tend to reduce nitrate uptake and enhance their own biological N fixation to meet N demand. In contrast, high nitrate concentrations inhibit nodulation and biological N fixation [4]. Studies on single-root soybean plants have shown that the contents of allantoinic acid and allantoin in stem xylem sap decrease after nitrate supply, while the content of asparagine (Asn) in the shoots increases. High concentrations of nitrate significantly inhibit nodule formation and nitrogenase activity in nodules, and the inhibitory effect becomes stronger with increasing nitrate concentration and duration of nitrate application [5,6,7]. In non-nodulated soybean plants, nitrate supply increases the Asn content in stem xylem sap, whereas the glutamine (Gln) content shows no significant change [8]. In split-root experiments, supplying nitrate to one half of the soybean root system inhibited biological N fixation on the nitrate-supplied side but promoted biological N fixation on the N-free side [9,10]. Compared with bilateral N-free treatments, nitrate supply to one half of the root system increased the contents of Asn and allantoin in soybean leaves, while the contents of Gln and allantoin decreased. When both halves of the root system were supplied with nitrate, the contents of Asn and Gln in soybean leaves increased [11,12]. In experiments using double-root soybean plants with unilateral nodulation, nitrate supplied to the non-nodulated root side promoted the accumulation of ureides in stems and nodules, inhibited nodule formation on the nodulated side, and reduced nodule nitrogenase activity [13,14,15]. Studies on N-containing compounds have shown that nitrate is assimilated in the roots of double-root soybean plants and is mainly transported in the form of Asn through the phloem and xylem, whereas the concentration of Gln remains relatively stable in the plant [16].

Nitrate absorbed by leguminous crops is reduced to ammonium through the action of nitrate reductase and nitrite reductase. The resulting ammonium subsequently enters the glutamine synthetase-glutamate synthase (GS-GOGAT) cycle to form Gln [17]. And some part of Gln is readily assimilated into Asn by Asparagin synthetase [18]. Injection of Asn into the phloem of alfalfa showed that, within 48 h after treatment, the increase in Asn concentration in nodules was accompanied by a decrease in nitrogenase activity, suggesting that Asn may act as a signaling molecule regulating biological N fixation in alfalfa nodules [19]. Studies on single-root bean plants have shown that after the application of Asn or Gln, seedling size and leaf area initially increased but subsequently decreased with increasing concentrations of Asn or Gln. High concentrations of Asn or Gln significantly increased the amide N concentration in bean plants [20,21]. In single-root soybean plants supplied with Asn, it was found that Asn may inhibit the hydrolysis of allantoinic acid by chelating Mn^2+^, resulting in the accumulation of allantoinic acid and a subsequent reduction in nitrogenase activity in nodules [22]. In addition, Asn may inhibit nodulation and biological N fixation by promoting the accumulation of Asn or ureide compounds in nodules [23]. Experiments applying nitrate and Gln to single-root soybean plants showed that both treatments reduced nodule weight and volume and inhibited nitrogenase activity, whereas replacing the Gln treatment with an N-free nutrient solution significantly increased nitrogenase activity in nodules [24].

At present, extensive studies have been conducted on the effects of nitrate supply on nodulation and biological N fixation in legumes. However, relatively few studies have examined the indirect effects of Asn and Gln supply on nodulation and biological N fixation. Moreover, it remains unclear whether nitrate, Asn, and Gln have synergistic effects on soybean nodulation and biological N fixation. To further clarify the relationships among nitrate, Asn, Gln, and soybean nodulation and biological N fixation, and to avoid the potential toxic effects caused by the direct contact of N sources with nodules, this study constructed a unilateral nodulated double-root soybean system by grafting nodulated and non-nodulated soybean plants. Nitrate, Asn, and Gln were supplied to the non-nodulated side to analyze the effects of different N sources on soybean nodulation and biological N fixation.

## 2. Results

### 2.1. Effect of Nitrate on Soybean Nodulation and Biological N Fixation

**Figure 1:**
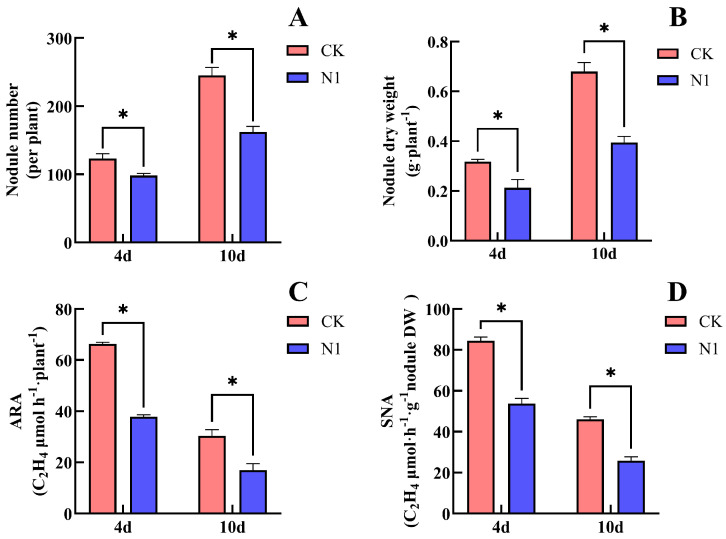
Effects of nitrate supply on the non-nodulated side on the nodule number, nodule dry weight and nitrogenase activity of soybean. (**A**) Nodule number; (**B**) nodule dry weight; (**C**) acetylene reduction activity (ARA); (**D**) specific nitrogenase activity (SNA). The (*) in the plot indicates that the difference between different treatments at the same time is significant at the 0.05 level. The number of replications “*n* = 4”. The error line indicates four repeated standard errors, and the following figure is the same.

Treatment with 14 mM nitrate significantly inhibited the number, dry weight, and nitrogenase activity of soybean nodules (Figure 1). After 4 days of nitrate (N1) treatment, compared with the CK, the nodule number decreased by 20.3%, nodule dry weight decreased by 34.4%, ARA decreased by 43.1%, and SNA decreased by 36.2%. All differences between N1 and CK were statistically significant. After 10 days of treatment, compared with CK, the nodule number in N1 decreased significantly by 33.9%, the nodule dry weight decreased markedly by 41.2%, ARA decreased significantly by 44.4%, and SNA decreased significantly by 44.0%. These results indicate that supplying nitrate to the non-nodulated side roots significantly inhibited nodule formation and nitrogenase activity in soybean nodules. Moreover, the inhibitory effect became more pronounced with increasing duration of nitrate supply.

### 2.2. Effect of Nitrate on the Content of N Compounds in Soybean

**Figure 2:**
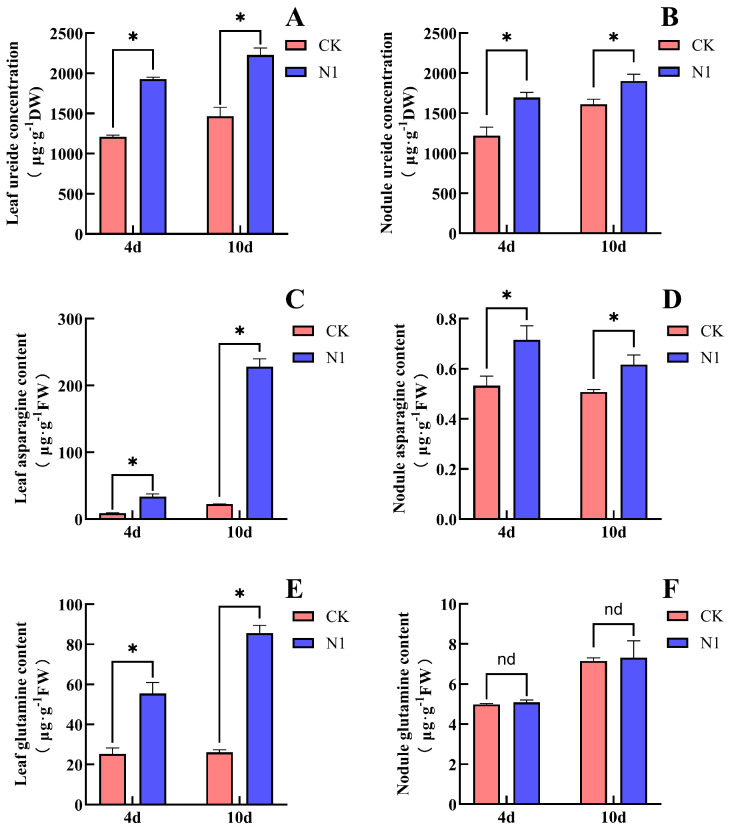
Effects of nitrate supply on the non-nodulated side on the contents of ureide, Asn and Gln in soybean leaves and nodules. (**A**) Leaf ureide content; (**B**) nodule ureide content; (**C**) leaf Asn content; (**D**) nodule Asn content; (**E**) leaf Gln content; (**F**) nodule Gln content. The (*) in the plot indicates that the difference between different treatments at the same time is significant at the 0.05 level, and the nd indicates that the difference between different treatments at the same time is not significant at the 0.05 level.

Treatment with 14 mM nitrate significantly promoted the accumulation of ureides and Asn in soybean leaves and nodules, as well as the accumulation of Gln in leaves (Figure 2). After N1 treatment, compared with the CK, the ureide content in leaves increased significantly by 55.5%, and the ureide content in nodules increased significantly by 39.0%. The Asn content in leaves increased significantly by 287%, and that in nodules increased markedly by 35.9%. The Gln content in leaves increased significantly by 119%, whereas the Gln content in nodules increased, but the difference was not statistically significant. After 10 days of treatment, compared with CK, the ureide content in leaves under N1 increased significantly by 52.0%, and that in nodules increased substantially by 18.0%. The Asn content in leaves increased significantly by 919%, while the Asn content in nodules increased markedly by 21.6%. The Gln content in leaves increased significantly by 228%, whereas the Gln content in nodules showed no significant change. These results indicate that nitrate promoted the accumulation of ureides in both soybean leaves and nodules, and the magnitude of this promotion decreased with increasing duration of nitrate supply. Nitrate also enhanced the accumulation of Asn in leaves and nodules; the increase in leaves became more pronounced with prolonged nitrate treatment, whereas the increase in nodules showed the opposite trend. With prolonged nitrate treatment, the accumulation of Gln in leaves gradually increased, while nitrate supply had no significant effect on Gln content in nodules. The relatively large standard errors observed in some results (Figure 2D,F) reflect biological variation among individual plants, as the grafting and nodulation process may introduce heterogeneity in nitrogen assimilation and transport.

### 2.3. Effects of Asn on Soybean Nodulation and Biological N Fixation

**Figure 3:**
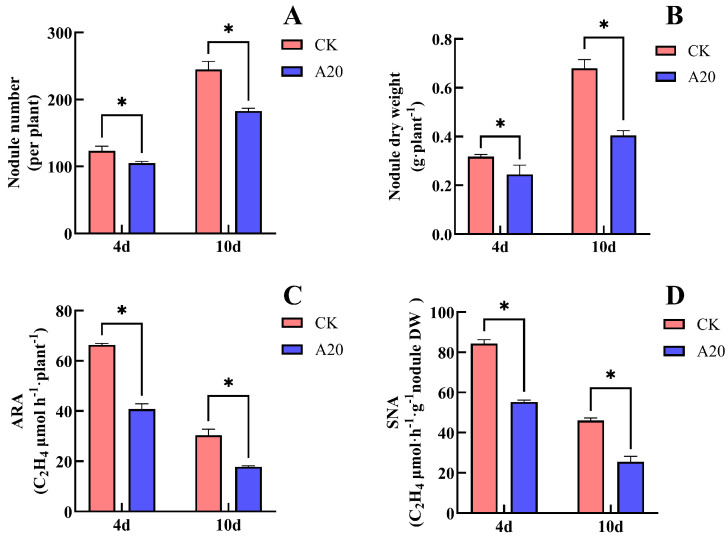
Effects of Asn supply on the non-nodulated side on the nodule number, nodule dry weight and nitrogenase activity of soybean. (**A**) Nodule number; (**B**) nodule dry weight; (**C**) acetylene reduction activity (ARA); (**D**) specific nitrogenase activity (SNA). The (*) in the plot indicates that the difference between different treatments at the same time is significant at the 0.05 level.

Treatment with 20 mM Asn significantly inhibited nodule number, nodule dry weight, and nitrogenase activity in soybean (Figure 3). After 4 days of Asn (A20) treatment, compared with the CK, nodule number decreased by 14.80%, nodule dry weight decreased by 21.9%, ARA decreased by 38.6%, and SNA decreased by 34.5%. All differences between A20 and CK were statistically significant. After 10 days of treatment, compared with CK, the nodule number in A20 decreased substantially by 25.4%, nodule dry weight decreased significantly by 39.7%, ARA decreased significantly by 41.2%, and SNA decreased significantly by 44.4%. These results indicate that supplying Asn to the non-nodulated side roots significantly inhibited soybean nodule formation and nodule nitrogenase activity. Moreover, the inhibitory effect became more pronounced with increasing duration of Asn supply.

### 2.4. Effect of Asn on the Content of N Compounds in Soybean

**Figure 4:**
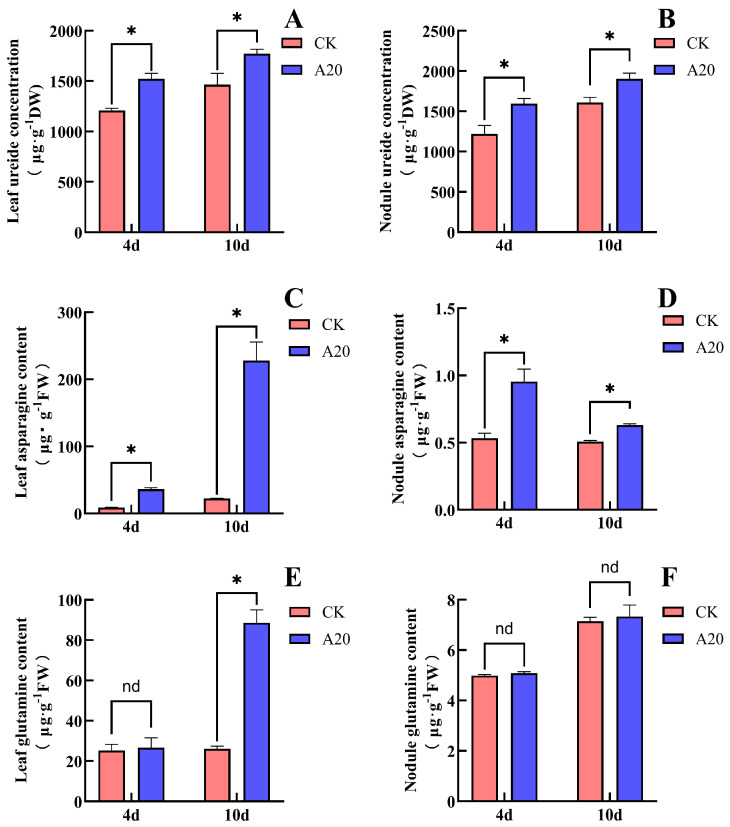
Effects of Asn supply on the non-nodulated side on the contents of ureide, Asn and Gln in soybean leaves and nodules. (**A**) Leaf ureide content; (**B**) nodule ureide content; (**C**) leaf Asn content; (**D**) nodule Asn content; (**E**) leaf Gln content; (**F**) nodule Gln content. The (*) in the plot indicates that the difference between different treatments at the same time is significant at the 0.05 level, and the nd indicates that the difference between different treatments at the same time is not significant at the 0.05 level.

Treatment with 20 mM Asn significantly affected the contents of N compounds in soybean leaves and nodules (Figure 4). After 4 days of Asn (A20) treatment, compared with the CK, the ureide content in leaves increased significantly by 33.4%, and the ureide content in nodules increased significantly by 30.7%. The Asn content in leaves increased significantly by 314%, and the Asn content in nodules increased substantially by 35.9%. The Gln contents in both leaves and nodules increased, but the differences were not statistically significant. After 10 days of treatment, compared with CK, the ureide content in A20 leaves increased substantially by 28.9%, and that in nodules increased significantly by 18.2%. The Asn content in leaves increased significantly by 919%, while the Asn content in nodules increased significantly by 23.5%. The Gln content in leaves increased significantly by 240%, whereas the Gln content in nodules showed no significant change. These results showed that Asn supply promoted the accumulation of ureides in soybean leaves and nodules, and the magnitude of this promotion decreased with increasing duration of Asn supply. Asn supply markedly increased Asn accumulation in leaves, and the magnitude of this increase became greater with prolonged treatment. In nodules, Asn accumulation was promoted in the short term but showed no significant change after prolonged supply. Short-term Asn supply had no significant effect on Gln content in either leaves or nodules, whereas long-term Asn supply promoted Gln accumulation in leaves but had no significant effect on Gln content in nodules. The relatively large standard errors observed in some results (Figure 4C–F) reflect biological variation among individual plants, as the grafting and nodulation process may introduce heterogeneity in nitrogen assimilation and transport.

### 2.5. Effects of Gln on Soybean Nodulation and Biological N Fixation

**Figure 5:**
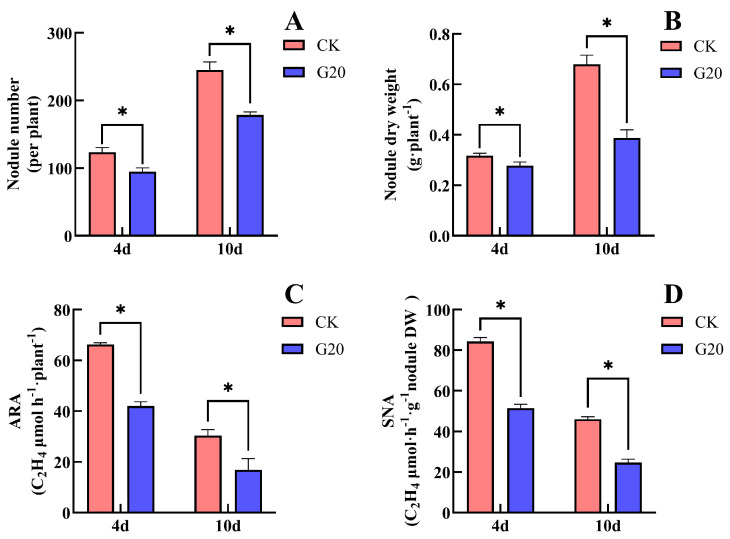
Effects of Gln supply on the non-nodulated side on the nodule number, nodule dry weight and nitrogenase activity of soybean. (**A**) Nodule number; (**B**) nodule dry weight; (**C**) acetylene reduction activity (ARA); (**D**) specific nitrogenase activity (SNA). The (*) in the plot indicates that the difference between different treatments at the same time is significant at the 0.05 level.

Treatment with 20 mM Gln significantly inhibited nodule number, nodule dry weight, and nitrogenase activity in soybean (Figure 5). After 4 days of Gln (G20) treatment, compared with the CK, nodule number decreased by 23.1%, nodule dry weight decreased by 12.5%, ARA decreased by 36.7%, and SNA decreased by 39.0%. All differences between G20 and CK were statistically significant. After 10 days of treatment, compared with CK, the nodule number in G20 decreased markedly by 27.1%, nodule dry weight decreased significantly by 42.7%, ARA decreased significantly by 44.2%, and SNA decreased markedly by 46.2%. These results showed that supplying Gln to the non-nodulated side roots significantly inhibited soybean nodule formation and nitrogenase activity. Moreover, the inhibitory effect became more pronounced with increasing duration of Gln supply.

### 2.6. Effects of Gln on the Content of N Compounds in Soybean

**Figure 6:**
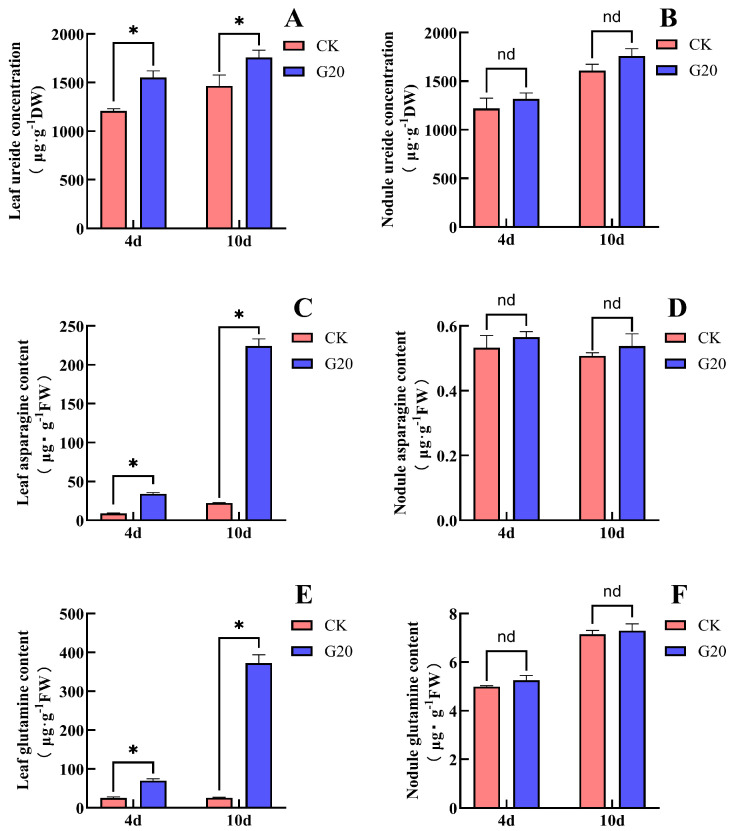
Effects of Gln supply on the non-nodulated side on the contents of ureide, Asn and Gln in soybean leaves and nodules. (**A**) Leaf ureide content; (**B**) nodule ureide content; (**C**) leaf Asn content; (**D**) nodule Asn content; (**E**) leaf Gln content; (**F**) nodule Gln content. The (*) in the plot indicates that the difference between different treatments at the same time is significant at the 0.05 level, and the nd indicates that the difference between different treatments at the same time is not significant at the 0.05 level.

Treatment with 20 mM Gln significantly promoted the accumulation of N compounds in soybean leaves (Figure 6). After 4 days of Gln (G20) treatment, compared with the CK, the ureide content in leaves increased significantly by 28.6%, the Asn content increased significantly by 290%, and the Gln content increased significantly by 118%. In contrast, no significant changes were observed in the contents of ureides, Asn, or Gln in nodules. After 10 days of treatment, compared with CK, the ureide content in G20 leaves increased significantly by 20.0%, the Asn content increased significantly by 902%, and the Gln content increased markedly by 1327%. Similarly, no significant changes were observed in any of these indices in nodules. These results indicated that Gln supply significantly promoted the accumulation of ureides, Asn, and Gln in soybean leaves. The promotive effect on ureide accumulation decreased with prolonged treatment time, whereas the promotive effects on Asn and Gln accumulation increased with longer treatment duration. In contrast, Gln supply had no significant effect on the contents of ureides, Asn, or Gln in nodules. The relatively large standard errors observed in some results (Figure 6D) reflect biological variation among individual plants, as the grafting and nodulation process may introduce heterogeneity in nitrogen assimilation and transport.

### 2.7. Correlation Between Asn Content in Soybean Leaves and Ureide Concentration in Nodules and Leaves

Pearson’s correlation analysis revealed that leaf Asn content was significantly positively correlated with both nodule ureide concentration (r = 0.760, *p* < 0.01) and leaf ureide concentration (r = 0.661, *p* < 0.01) (Table 1). These results indicate that Asn accumulation is closely associated with ureide accumulation in both nodules and leaves.

### 2.8. Correlation Between Gln Content in Soybean Leaves and Ureide Concentration in Nodules and Leaves

Pearson’s correlation analysis was performed to examine the relationship between leaf Gln content and ureide accumulation. The results showed that leaf Gln content was not significantly correlated with nodule ureide concentration (r = 0.327, *p* > 0.05) or leaf ureide concentration (r = 0.257, *p* > 0.05) (Table 2). The relatively low correlation coefficients indicate that Gln accumulation is not closely associated with ureide accumulation in either nodules or leaves.

## 3. Discussion

### 3.1. Effects of Nitrate, Asn, and Gln on Nodulation and Biological N Fixation

Both direct and indirect nitrate supply can inhibit nodulation and biological N fixation in leguminous crops. Previous studies have shown that nodule formation is significantly inhibited when nitrate concentrations exceed 10 mM [25]. In experiments where soybean roots were divided into upper and lower layers, the application of nitrate to the lower roots significantly inhibited nodule growth and nitrogenase activity in the upper roots [26]. In the present study, unilateral nodulated double-root soybean plants were constructed using a grafting method. Indirect nitrate supply to the non-nodulated side roots resulted in inhibition of nodule number, nodule dry weight, and nitrogenase activity (ARA and SNA) [13,14,15,16,27]. Consistent with these findings, our results showed that the number of nodules, nodule dry weight, and nitrogenase activity of unilateral nodulated double-root soybean plants were significantly reduced after 4 and 10 days of indirect nitrate supply (Figure 1), which is consistent with previous reports.

Previous studies have reported that the direct supply of Asn or Gln to soybean or broad bean plants produces effects similar to those of nitrate, including reductions in nodule weight, number, and volume, as well as inhibition of nodule nitrogenase activity [24,28,29,30]. However, because Asn and Gln are high-quality amino acid sources of both N and carbon, their direct application to soybean roots may stimulate the proliferation of rhizosphere microorganisms (such as bacteria and fungi), thereby affecting nodule health and interfering with the accuracy of experimental results. To minimize this interference, some studies have applied antibiotics and antifungal solutions together with Asn and Gln treatments [23]. However, such approaches cannot exclude the potential effects of these antimicrobial agents on nodule growth and biological N fixation. In the present study, unilateral nodulated double-root soybean plants were used, and Asn and Gln were supplied only to the non-nodulated side roots. This approach eliminated the potential “toxic” effects caused by direct contact of these compounds with nodules. Our results showed that both Asn and Gln still inhibited soybean nodulation and biological N fixation even without direct contact with nodules (Figure 3 and Figure 5). These findings suggest that the effects of Asn and Gln on the N-fixation capacity of soybean nodules are also systemic.

### 3.2. Asn as a Signaling Substance Regulating Soybean Nodulation and Biological N Fixation

Ureides are mainly derived from root nodules. On the one hand, ureides are transported to the shoots to provide N nutrition for plant growth; on the other hand, ureide concentration can regulate the N-fixation capacity of nodules [4,14]. Proteomic and metabolomic analyses have shown that the inhibition of nodulation and biological N fixation by exogenous N involves multiple metabolic pathways, including N metabolism, amino acid metabolism, and various signaling molecules such as Asn and nitric oxide (NO) [31]. Ishikawa et al. conducted a transcriptome and metabolome analysis of soybean roots and nodules treated with 5 mM nitrate for 24 h. In the nodules, they observed that asparagine synthetase (AS) was significantly upregulated, while asparaginase was downregulated, leading to a marked accumulation of Asn (3.3-fold). In contrast, the increase in Gln was relatively modest (1.6-fold), and the expression of Gln-related enzymes was not significantly altered. Although ureides also accumulated in the nodules, the authors attributed this to decreased ureide transport rather than enhanced synthesis, as the expression of ureide biosynthetic enzymes was not upregulated. These findings suggest that under nitrate treatment, Asn accumulation may precede and potentially drive ureide accumulation [32]. Bacanamwo and Harper reported that the Asn content in the shoots of soybean increased sharply within 24 h after the addition of 15 mM nitrate to the hydroponic nutrient solution, whereas little accumulation of Asn occurred in the nodules. They suggested that Asn may be transported to nodules through the phloem as an N signal, thereby inhibiting nitrogenase activity [33]. Wang et al. found that nitrate supply to soybean was accompanied by the accumulation of Asn in shoots (including leaves, stems, and petioles), the accumulation of ureides in nodules, and the decrease in uric acid oxidase and nodule nitrogenase activity in nodules. They proposed that nitrate inhibits soybean nodulation and biological N fixation by suppressing uric acid oxidase activity through Asn and promoting ureide accumulation in shoots [14]. Ono et al., through hydroponic experiments with soybean, found that treatment with 5 mM NaNO_3_ reduced the contents of ureides and Gln in nodules while increasing the content of Asn. They suggested that after nitrate supply, Asn replaces ureides as the major form of N transport [34]. Yamashita et al. further reported that Gln is relatively unstable in soybean and can be readily converted to Asn for transport [24]. Early ^15^N-tracer studies further elucidated the distinct roles of Asn and Gln in nitrogen transport: Asn was identified as the primary nitrogen compound synthesized and transported to the shoots following nitrate absorption, whereas Gln played only a secondary role. Moreover, these studies demonstrated that ureides are the predominant form of nitrogen exported from nodules to the shoots, with the amount of ^15^N derived from ^15^N_2_ fixation being 10–50 times higher than that derived from ^15^NO_3_^−^ [35]. In the present study, nitrate supplied to the non-nodulated side roots significantly promoted the accumulation of ureides, Asn, and Gln in soybean leaves, and similar effects were observed when Asn or Gln was supplied. Among these treatments, Asn accumulated rapidly after Gln supply, whereas Gln accumulation was observed only after 10 days of Asn supply (Figure 2, Figure 4 and Figure 6). These results indicate that Gln can be rapidly converted into Asn in leaves, whereas the conversion of Asn into Gln occurs more slowly. Asn appears to be the major N transport compound in soybean leaves. The supply of nitrate and Asn promoted the accumulation of ureides and Asn, whereas the Gln content did not increase significantly. In contrast, after Gln supply, the contents of ureides, Asn, and Gln in nodules did not change significantly (Figure 2, Figure 4 and Figure 6). This suggests that Gln may mainly serve as an N source rather than a primary regulatory signal in nodulation and biological N fixation. Correlation analysis further supports this inference. Leaf Asn content was significantly positively correlated with both nodule ureide concentration and leaf ureide concentration (Table 1), indicating that Asn accumulation is closely associated with ureide accumulation. In contrast, leaf Gln content showed no significant correlation with either nodule ureide concentration or leaf ureide concentration (Table 2), indicating that Gln accumulation is not associated with ureide accumulation. Furthermore, the accumulation of Asn and ureides in nodules occurred concurrently with the inhibition of nodulation and biological N fixation. Asn and Gln are the major amino acids involved in N transport in soybean and are derived from both nodule synthesis and the conversion of ureides in shoots [36]. Therefore, we propose that the inhibitory effect of nitrate on soybean nodulation and biological N fixation is associated with the accumulation of ureides in both leaves and nodules. The accumulation of ureides in these organs is likely caused by increased Asn levels in leaves and nodules, which may limit the further assimilation of ureides into Asn. When ureide levels increase within the plant, nodules may receive a signal indicating that N supply is sufficient, thereby reducing the N-fixation capacity. In this experiment, in contrast to the findings of Ono et al. and Ishikawa et al., we did not observe a decrease in the contents of ureides, Asn, or Gln, nor did we detect a clear temporal sequence between Asn and ureide accumulation under nitrate treatment [32,34]. This discrepancy may be attributed to the slower transformation and transport of nitrogen-containing compounds in the double-root soybean system, or to differences in tissue distribution preferences compared with single-root soybeans. Future studies could further investigate the transformation and transport dynamics of nitrate, Asn, and Gln among different organs by extending the treatment duration or employing isotope labeling approaches. Interestingly, the signaling role of Asn may not be limited to legume nodules. In maize, Asn derived from endosperm hydrolysis was found to significantly inhibit the induction of nitrate reductase in young seedlings, with root tissues being particularly sensitive [37]. Similarly, in Chinese cabbage, when Asn was used as a partial substitute for nitrate, it reduced nitrate uptake by suppressing the expression of nitrate transporters [38]. Although these studies were conducted in non-leguminous species, they collectively suggest that Asn can function as a regulatory molecule beyond its role as a nitrogen carrier, consistent with our hypothesis that Asn acts as a systemic signal in the inhibition of nodulation and nitrogen fixation.

This study has several limitations. First, the experiments were conducted using specific concentrations and a single treatment duration (4 d and 10 d), which may not fully capture the dose-dependent and temporal dynamics of the systemic response. Second, while the grafting system effectively isolates systemic effects, the grafting process itself may introduce physiological stress that could influence nodulation. Third, the proposed role of Asn as a signaling molecule is based primarily on content analysis; direct evidence is still lacking. Future studies should employ genetic approaches, such as using asparagine synthetase or asparaginase mutants, to directly test the causal role of Asn in mediating nitrate-induced inhibition.

## 4. Materials and Methods

The experiment was conducted in 2025 at the experimental base of Northeast Agricultural University using a sand culture system. The nodulated soybean variety used in this study was Kennong 30 (obtained from the College of Plant Science and Technology, Heilongjiang Bayi Agricultural University, Harbin, China), while the non-nodulated soybean varieties were WDD01795, L8-4858 (obtained from the Chinese Academy of Agricultural Sciences, Beijing, China). Plastic buckets with an upper diameter of 30 cm, a bottom diameter of 20 cm, and a height of 28 cm were used as culture containers. Each bucket was divided into two equal compartments by a plastic partition placed in the middle. Two round holes with a diameter of approximately 1 cm were drilled at the bottom of the bucket to allow drainage. The junction between the bucket and the partition was sealed with tape to prevent the nutrient solutions in the two compartments from mixing. Each compartment was filled with 10 kg of clean sand.

### 4.1. Experimental Design

**Figure 7:**
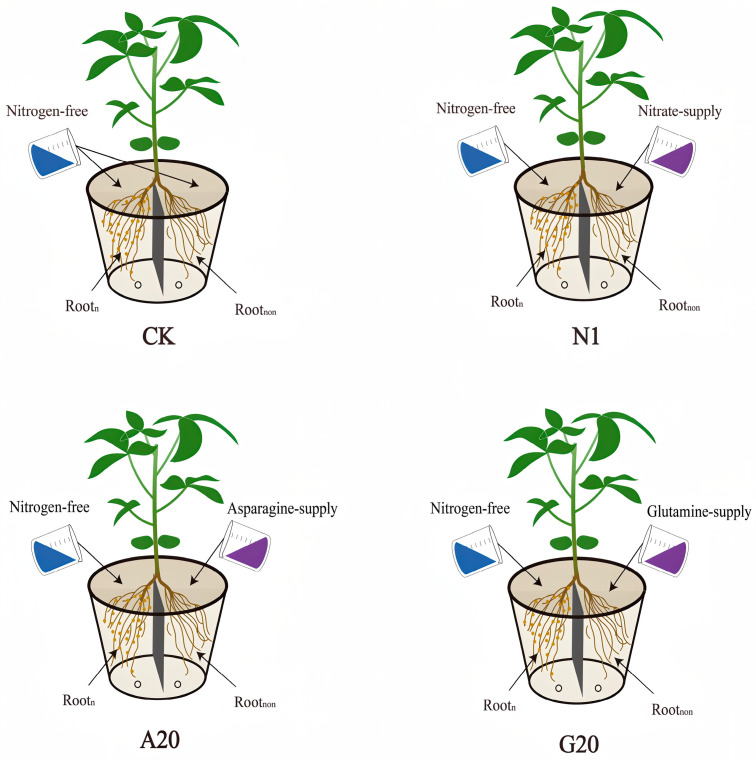
Schematic diagram of test treatment method. Unilateral nodulation double-root soybean plants were prepared by grafting. During the treatment period, the nodulated side roots (Root-n) were continuously supplied with nitrogen-free nutrient solution, while the non-nodulated side roots (Root-non) were supplied with different nitrogen sources: nitrate (14 mM, designated as N1), asparagine (20 mM, designated as A20), or glutamine (20 mM, designated as G20). CK: control treatment with both sides supplied with nitrogen-free nutrient solution.

The method described by Liu et al. was used to construct unilateral nodulated double-root soybean plants and to prepare the N-free nutrient solution [39]. Soybean nodules preserved from the previous year were ground and mixed with water, and the supernatant was collected, ensuring that each liter contained approximately 5 g of nodules. Before the true leaves were fully expanded, the grafted seedlings were inoculated with the supernatant on both sides every morning for 7 consecutive days. From the VC to V4 stage, the grafted seedlings were supplied once daily in the morning with a nutrient solution containing 1 mM KNO_3_ on both sides. After the V4 stage, both sides of the double-root soybean plants were supplied with N-free nutrient solution once daily in the morning until the R1 stage, resulting in a 6-day N starvation treatment. At the R1 stage, nutrient solutions were applied twice daily, in the morning and evening. The non-nodulated side roots were supplied with different N sources: 14 mM KNO_3_ (designated as N1), 20 mM Asn (designated as A20), or 20 mM Gln (designated as G20). (In the preliminary stage of this study, we used 7 mM KNO_3_ [40]. Considering that nitrogen transport in the dual-root system may exhibit a lag effect, we increased the KNO_3_ concentration to 14 mM in subsequent experiments to achieve a more pronounced inhibitory effect [13,15,27]. Regarding Asn and Gln, most previous studies have used concentrations ≤ 10 mM. However, we considered that the inhibitory effect under indirect supply conditions might be less evident, so we set the concentration of Asn and Gln to 20 mM [20,21,23,29]). The nodulated lateral roots of all treatments received only N-free nutrient solution. Each application consisted of 250 mL of nutrient solution per side. The control treatment (CK) received N-free nutrient solution on both sides of the root system (Figure 7). Each treatment had eight replicates. During sampling, four replicates were harvested by cutting the plants at the grafting site. The third fully expanded trifoliate leaf from the top was collected. The nodulated lateral roots were retained, washed with distilled water, and gently dried with filter paper. Nodule nitrogenase activity was measured immediately. The nodules were then removed to determine nodule number and subsequently dried together with the leaves in an oven at 65 °C until constant weight. The ureide content in leaves, nodule dry weight, and ureide content in nodules were determined. For the remaining four replicates, the third fully expanded trifoliate leaves and all nodules were collected, rinsed with distilled water, wrapped in tin foil, and stored in a laboratory refrigerator for the determination of Asn and Gln contents in leaves and nodules.

### 4.2. Determination Method

Nodule nitrogenase activity, including acetylene reduction activity (ARA) and specific nitrogenase activity (SNA), was determined by the acetylene reduction method [41]; ureide content in leaves and nodules was measured by the glyoxylic acid-phenylhydrazine colorimetric method [42]; the contents of Asn and Gln in leaves and nodules were determined by the phenyl isothiocyanate (PITC) derivatization method combined with high-performance liquid chromatography [16].

### 4.3. Data Analysis

Microsoft Excel 2019 was used for data organization and recording. Statistical analyses were performed using SPSS 26.0 (IBM Corp., Armonk, NY, USA). Differences between treatment groups and the control were analyzed using an independent-samples *t*-test. Differences were considered statistically significant at *p* < 0.05. The values of each parameter represent the mean of four replicates. All figures were generated using GraphPad Prism 8 (GraphPad Software Inc., San Diego, CA, USA).

## 5. Conclusions

The supply of nitrate, Asn, and Gln to the non-nodulated side roots of unilateral nodulated double-root soybean plants inhibited nodule formation and nitrogenase activity. In soybean plants, nitrate is assimilated into Asn and Gln. Asn accumulates in leaves and nodules and suppresses nodulation and biological N fixation by promoting ureide accumulation, thereby transmitting a signal of sufficient N status within the plant. In contrast, Gln appears to function mainly as an intermediate metabolite rather than a signaling substance in the regulation of soybean nodulation and biological N fixation.

## Figures and Tables

**Table 1:** Pearson’s correlation of leaf asparagine content with nodule ureide concentration and leaf ureide concentration of soybean nodules.

Traits	Leaf Asparagine Content
Nodule ureide concentration	0.760 **
Leaf ureide concentration	0.661 **

Note: “**” represents *p* < 0.01.

**Table 2:** Pearson’s correlation of leaf glutamine content with nodule ureide concentration and leaf ureide concentration of soybean nodules.

Traits	Leaf Glutamine Content
Nodule ureide concentration	0.327 nd
Leaf ureide concentration	0.257 nd

Note: “nd” represents *p* > 0.05.

## Data Availability

The original data presented in this study are included in the article.
